# Integron architecture and virulence-associated gene profiles in extensively drug-resistant high-risk *Pseudomonas aeruginosa* ST309 and ST233

**DOI:** 10.3389/fmicb.2026.1833035

**Published:** 2026-07-23

**Authors:** José Luis Fernández-Vázquez, Ma Dolores Jarillo-Quijada, Santiago Castillo-Ramírez, María Luisa Hernández-Medel, Manuelita Zavala-Pineda, Mónica Osorio-Guzmán, José Ignacio Santos-Preciado, María Dolores Alcántar-Curiel

**Affiliations:** 1 Laboratorio de Infectología, Microbiología e Inmunología Clínicas, Unidad de Investigación en Medicina Experimental, Facultad de Medicina, Universidad Nacional Autónoma de México. Ciudad de México, Mexico; 2 Programa de Genómica Evolutiva, Centro de Ciencias Genómicas, Universidad Nacional Autónoma de México, Cuernavaca, Morelos, Mexico; 3 Unidad de Infectología, Hospital General de México Dr. Eduardo Liceaga. Ciudad de México, Mexico; 4 Unidad de Infectología, Hospital General de León, León, Guanajuato, Mexico

**Keywords:** *Pseudomonas aeruginosa*, chromosomal resistance regions, class 1 integrons, ExoU/ExoS virulotypes, extensively drug-resistant, high-risk clones, ST233, ST309

## Abstract

**Background:**

*Pseudomonas aeruginosa* is a major cause of healthcare-associated infections and exhibits considerable genomic plasticity, which allows the accumulation and structural organization of resistance determinants while retaining virulence-associated traits in some high-risk clones (HRCs). HRCs such as Sequence Type (ST) 309 and ST233 are increasingly associated with extensively drug-resistant (XDR) and pandrug-resistant (PDR) phenotypes worldwide. However, the integron architecture and virulome associated with XDR *P. aeruginosa* have not been fully characterized. This study aimed to characterize the resistome and virulome of highly resistant ST309 and ST233 isolates and to analyze the structural variability of chromosomal resistance regions.

**Methods:**

Whole-genome sequencing and comparative genomic analyses were performed on four bloodstream *P. aeruginosa* isolates recovered from two tertiary-care hospitals in Mexico.

**Results:**

Three isolates (Psa/12, Psa/19, and Psa/21) were classified as XDR and belonged to ST309, whereas one isolate (Psa/20) was PDR and assigned to ST233. The ST233 isolate carried the highest number of resistance genes, including the carbapenemase gene *bla*_*VIM–2*_. ST309 isolates carried between 11 and 12 resistance genes and shared a conserved class 1 integron-associated chromosomal resistance region flanked by *int1* and insertion sequences, with structural differences among the analyzed isolates. In contrast, ST233 carried a multidrug resistance region highly similar to transposon Tn6061. The *in silico* virulome analysis identified a large, conserved repertoire of genes (203–209 genes), with 199 genes shared across all isolates. Notably, 14 virulence-associated genes differed between the ST309 and ST233 isolates, including differential carriage of type III secretion system effectors (*exoU* in ST309 and *exoS*/*exoY* in ST233), phenazine biosynthesis genes, and type IV pili components.

**Conclusion:**

XDR high-risk *P. aeruginosa* ST309 and ST233 isolates carried distinct chromosomal resistance regions and showed differences in virulence genes. The analyzed isolates combined extensive antimicrobial resistance with a largely conserved set of virulence-associated genes, highlighting the persistence of clinically relevant high-risk clones in Mexican hospitals.

## Introduction

In 2017, the World Health Organization classified carbapenem-resistant *Pseudomonas aeruginosa* as a critical priority pathogen for research and development of new antibiotics (Antimicrobial Resistance Collaborators, 2022; Tacconelli et al., 2018). One of the most clinically significant opportunistic pathogens in the world, *P. aeruginosa* is a Gram-negative, aerobic, non-fermenting bacillus which is frequently detected in environmental reservoirs. It primarily affects immunocompromised individuals, but it is also frequently associated with bloodstream infections, intra-abdominal or peritoneal infections, lower respiratory tract infections, and cystic fibrosis (Papa-Ezdra et al., 2024; Agodi et al., 2007; Thuong et al., 2003). The increasing prevalence of resistance to carbapenems and other last-resort antimicrobial agents has further complicated the treatment and control of *P. aeruginosa* infections, reinforcing their role as a major cause of healthcare-associated infections, particularly those associated with surgical sites and medical devices (Kracalik et al., 2022).

Given the increasing diversity of antimicrobial resistance profiles in *P. aeruginosa*, standardized definitions for multidrug-resistant (MDR), extensively drug-resistant (XDR), and pandrug-resistant (PDR) categories have been adopted to facilitate the interpretation and comparison of resistance patterns across studies (Magiorakos et al., 2012; Sastre-Femenia et al., 2023). Infections with MDR and XDR strains of *P. aeruginosa* have limited treatment options and a higher mortality rate (Kawalek et al., 2020), emphasizing the need to characterize the genomic architecture associated with XDR phenotypes in high-risk clones.

Antimicrobial resistance in *P. aeruginosa* is found to be caused by a small number of high-risk clones (HRCs) widely distributed (Hu et al., 2021; Gravningen et al., 2022). According to Oliver et al. (2015), Del Barrio-Tofiño et al. (2020), these include ST235, ST111, ST233, ST244, ST357, ST308, ST175, ST277, ST654, and ST298. Recently, ST309 has been proposed as an emerging HRC due to its international distribution and association with multidrug resistance determinants (Fonseca et al., 2022; Morales-Espinosa et al., 2017). HRCs are frequently linked to extended-spectrum β-lactamases (ESBLs), metallo-β-lactamases (MBLs), class 1 integrons, and specific serotypes—particularly O11 and O6—as well as type III secretion system (T3SS) effector profiles commonly reported in specific high-risk lineages, such as ExoU or ExoS (Yoon et al., 2017; Del Barrio-Tofiño et al., 2020).

Besides the acquisition of carbapenemases, *P. aeruginosa* may develop resistance to carbapenems through chromosomal mechanisms, including AmpC β-lactamase derepression, overexpression of efflux pumps, and loss or modification of the outer membrane porin OprD (Botelho et al., 2019). The accumulation and organization of resistance determinants within mobile genetic elements is consistent with the considerable genomic plasticity of *P. aeruginosa*, which possesses a relatively large genome for a bacterial pathogen (approximately 5–7 Mb) (Jurado-Martín et al., 2021). Under sustained antimicrobial pressure, HRCs circulating in healthcare settings can accumulate multiple resistance determinants while maintaining virulence-associated traits.

Carbapenem resistance in *P. aeruginosa* isolates from Mexico ranges from 24% to 48%, while ceftazidime and amikacin resistance ranges from 12% to 33%, as reported by the One Health Trust’s ResistanceMap database (One Health Trust, 2025. Date accessed: 25 February 2026). According to national reports, *P. aeruginosa* is one of the most common causes of infections associated with healthcare (González-Olvera et al., 2019), highlighting its clinical and epidemiological significance. In addition, in line with recent reports from Mexico, ST309 is an HRC that may have adapted and is disseminating in hospital settings (Torres-Rodríguez et al., 2026).

This study investigated the resistome and virulome of four highly resistant *P. aeruginosa* clinical isolates recovered in Mexico and analyzed the structural variability of chromosomal resistance regions in ST309 and ST233 HRCs associated with XDR phenotypes.

## Materials and methods

### Study design, clinical setting, and bacterial isolates

Four non-duplicate *P. aeruginosa* isolates were recovered from bloodstream infections in patients with healthcare-associated infections (HAIs) from two tertiary-care hospitals located in distinct geographic regions of Mexico. Three isolates (Psa/19, Psa/20, and Psa/21) were obtained from the Hospital General de México “Dr. Eduardo Liceaga” (HGM), Mexico City, and one isolate (Psa/12) from the Hospital General de León (LON), Guanajuato, Mexico. Isolates were cultured overnight in Luria–Bertani (LB) broth at 37 °C and preserved in 20% glycerol stocks at −70 °C until further analysis. Only one isolate per patient was included in the study.

### Ethics statement

The clinical isolates analyzed in this study were previously included in a published investigation approved by the Research and Ethics Committee of the Hospital General de México “Dr. Eduardo Liceaga” (approval number DI/20/405/03/10) (Alcántar-Curiel et al., 2025). All isolates were collected as part of routine clinical care and were irreversibly anonymized before research use. The present work constitutes a whole-genome sequencing and comparative genomic analysis of previously approved bacterial isolates. No additional patient recruitment or identifiable clinical data were involved. Therefore, no new ethical approval was required according to institutional and national regulations.

### Antimicrobial susceptibility testing

To characterize antimicrobial susceptibility profiles of isolates, antimicrobial susceptibility testing was performed using the automated VITEK^®^-2 system (bioMérieux). To characterize colistin susceptibility, minimum inhibitory concentrations (MIC) were determined according to Clinical and Laboratory Standards Institute (CLSI) standards by broth microdilution (Lewis and Pharm, 2025). Quality control for *E. coli* ATCC 25922 and *P. aeruginosa* ATCC 27853 were used (Rosales-Reyes et al., 2020). The definitions of the MDR, XDR, and PDR phenotypes were those described by Magiorakos et al. (2012).

### Pulsed-field gel electrophoresis

Genotypic relatedness was evaluated by pulsed-field gel electrophoresis (PFGE) as previously described (Alcántar-Curiel et al., 2025). Briefly, Samples were embedded in 1% agarose plugs, and genomic DNA was digested overnight with *BcuI* (Invitrogen, now Thermo Fisher Scientific MA, United States), according to the manufacturer’s instructions. The digested DNA samples were subjected to PFGE using a Gene Path System (Bio-Rad^®^ Hercules CA, United States) with the Gene Path Program Upgrade Kit Version 1.0 (DOS-based software). Pulsotype (PT) assignment was performed manually by constructing a binary matrix of band presence or absence in the electrophoretic profiles of the isolates, according to Tenover’s criteria (Tenover et al., 1995). Similarity between profiles was calculated from the binary matrix using the unweighted pair group method with arithmetic mean (UPGMA) and the Dice coefficient (Dice, 1945) with PAST software version 4.45 (Hammer et al., 2001). Isolates showing ≥ 85% similarity were considered to belong to the same pulsotype.

### Detection of structural variations in resistance regions

Confirmation of the length of the variable regions carrying resistance genes in ST309 isolates was performed by endpoint PCR. Specific primers were designed using Primer3 (Alcántar-Curiel et al., 2019). The primers used were PsaFw (5′ GGTCAAGTTATTACACAACTCATCCTGAGC-3′) and PsaRv (5′-ACGTCGGTTCGAGATGGCGCTCGATG-3′), which amplify the regions located between the *bla*_GES–20_ and *aadA1* genes. PCR amplification reactions were performed using GoTaq Green Master Mix (Promega, Madison, WI, United States) under standard conditions consisting of an initial denaturation at 95 °C for 5 min, followed by 35 cycles of 95 °C for 1 min, 58 °C for 1 min and 72 °C for 90 s, with a final extension of 72 °C for 15 min. Amplification products were resolved on 1% agarose gels.

### Whole-genome sequencing and bioinformatic analysis

Genomic DNA was extracted from overnight LB cultures using the Quick-DNA™ Fungal/Bacterial Miniprep Kit (Zymo Research, Irvine, CA, United States), following the manufacturer’s instructions. Agarose gel electrophoresis was used to assess the purity of DNA. An Illumina MiSeq platform (2 × 250 bp paired-end reads; ∼100 × coverage) was used for whole-genome sequencing at the Instituto Nacional de Medicina Genómica (INMEGEN), Mexico City (Fernández-Vázquez et al., 2023). SPAdes v3.13.1 was used to assemble the reads de novo for each genome (Bankevich et al., 2012). The quality of the genome assemblies was assessed using QUAST v5.2.0 (Mikheenko et al., 2023). Prokka v1.14.6 was used to annotate the genome (Seemann, 2014). SpeciesFinder 2.0 was used to confirm the species identification (Larsen et al., 2014). The MLST technique from PubMLST was used to assess sequence type (ST) *in silico* (Jolley et al., 2018). The *P. aeruginosa* serotyper PAst 1.0 was used to designate serotypes (Thrane et al., 2016; Camacho et al., 2009). To determine the genomic similarity across isolates and assess the genetic relatedness of isolates, pairwise average nucleotide identity (ANI) values were calculated from the assembled genomes of all isolates using ANI Calculator (Yoon et al., 2017) and default settings.

### Identification of resistance and virulence genes

The identification of acquired antimicrobial resistance genes (ARGs) was performed using ResFinder (v4.6.0) with default parameters, applying a minimum sequence identity threshold of 80% and a minimum coverage length of 60% and CARD 4.0.1 with the following recommended parameters: DNA sequences; only perfect and strict hits were considered; the “nudge” option for promoting loose hits to strict hits (≥95% identity) was excluded; and only high-quality sequences with sufficient coverage were included (Bortolaia et al., 2020; Alcock et al., 2023). Virulence-associated genes were identified using the Virulence Factor Database (VFDB 2.0) (Darmancier et al., 2022) with *P. aeruginosa* PA01 used as the reference genome. Plasmid replicons were identified using MOB-suite v3.0.3 and PlasmidSPAdes v3.11.1 (Antipov et al., 2016; Robertson and Nash, 2018; Robertson et al., 2020). Mobile genetic elements were identified using Mobile Element Finder v1.0.3 (Johansson et al., 2021), and prophage-related sequences were identified using PHASTEST v3.0 (Wishart et al., 2023).

The identification of genetic variants of genes associated with colistin resistance (*phoPQ, pmrAB, nfeD, oprH*) was performed by gene alignment using the Molecular Evolutionary Genetics Analysis MEGA 11.0.13 program (Tamura et al., 2021). The identification of predicted OprD homologs involved in colistin resistance was performed manually by inspecting the Prokka annotation files generated from the assemblies of the four *P. aeruginosa* strains, as well as the reference strain PAO1 genome (AE004091.2). Open reading frames (ORFs) corresponding to OprD were retrieved from each annotation file and analyzed while preserving the nomenclature assigned by Prokka, followed by the corresponding ORF amino acid (aa) length. Sequence alignments and comparative analyses were performed using Clustal Omega version 1.2.4 (Madeira et al., 2024).

### Statistical analysis

No inferential statistical comparisons were performed due to the exploratory and descriptive genomic nature of the study.

## Results

### Clinical origin and antimicrobial susceptibility profiles

The four *P. aeruginosa* isolates analyzed in this study were recovered from patients with healthcare-associated bloodstream infections. Isolate Psa/12 was obtained from the general surgery ward of the LON, whereas isolates Psa/19, Psa/20, and Psa/21 were recovered from the internal medicine ward, emergency department, and intensive care unit of the HGM, respectively. Regarding the clinical background, isolate Psa/12 was isolated from a patient with abdominal sepsis, isolate Psa/19 from a patient diagnosed with COVID-19, isolate Psa/21 from a patient with necrotizing fasciitis of the pelvic limb involving the gluteal, lumar and pelvic regions, while Psa/20 was recovered from a patient presenting with a sub-hepatic abscess associated with abdominal sepsis.

Antimicrobial susceptibility testing revealed marked heterogeneity among the isolates (Table 1). Psa/12, Psa/19, and Psa/21 were classified as XDR. Psa/12 and Psa/21 retained susceptibility to amikacin and cefepime, respectively, and both exhibited intermediate susceptibility to polymyxin B. In contrast, isolate Psa/19 displayed intermediate susceptibility only to gentamicin and resistance to the remaining agents evaluated. Psa/20 exhibited a PDR phenotype. Notably, all isolates showed high-level resistance to carbapenems (imipenem and meropenem; MIC ≥ 128 μg/mL) and fluoroquinolones. Furthermore, all four were resistant to colistin—one of the last-line therapeutic options for XDR infections—showing high clinically relevant concentrations (MIC = 4 μg/mL).

**TABLE 1:** Antimicrobial susceptibility profiles of *Pseudomonas aeruginosa* ST309 and ST233 clinical isolates.

No. isolate - hospital	ST*^b^*/serotype	AMK	GEN	PIP	CAZ	FEP	CIP	LVX	IMI	MEM	COL	POL	Clasification	Outcome
		MIC μg/mL*^a^*												
Psa/12 - LON*^c^*	309/O11	4 (S)	16 (R)	>256 (R)	128 (R)	>128 (R)	16 (R)	32 (R)	>128 (R)	>128 (R)	4 (R)	2 (I)	XDR	Improvement
Psa/19 - HGM*^d^*	309/O11	128 (R)	8 (I)	256 (R)	128 (R)	>128 (R)	16 (R)	32 (R)	128 (R)	128 (R)	4 (R)	4 (R)	XDR	Improvement
Psa/20 - HGM*^d^*	233/O6	>128 (R)	>128 (R)	256 (R)	32 (R)	128 (R)	16 (R)	32 (R)	>128 (R)	128 (R)	4 (R)	4 (R)	PDR	Improvement
Psa/21 - HGM*^d^*	309/O11	128 (R)	>128 (R)	256 (R)	64 (R)	8 (S)	16 (R)	64 (R)	>128 (R)	>128 (R)	4 (R)	2 (I)	XDR	Death

*^a^*Minimum inhibitory concentration (MIC).

*^b^*Sequence type.

*^c^*Hospital General de León, Guanajuato.

*^d^*Hospital General de México “Dr. Eduardo Liceaga. S, susceptible; I, intermediate; R, resistant; XDR, extensively drug-resistant; PDR, pandrug resistant. AMK, amikacin; GEN, gentamicin; PIP, piperacillin; CAZ, ceftazidime; FEP, cefepime; CIP, ciprofloxacin; LVX, levofloxacin; IMI, imipenem; MEM, meropenem; COL, colistin; POL, polymyxin B.

### Molecular typing and clonal diversity

**FIGURE 1:**
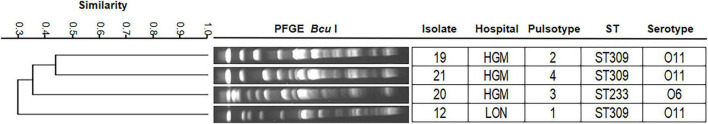
Pulsed-field gel electrophoresis (PFGE) patterns of *Pseudomonas aeruginosa* clinical isolates. Dendrogram analysis showing the genetic relatedness among isolates based on PFGE profiles. Sequence type (ST) is indicated for each strain. HGM, Hospital General de México; LON, Hospital General de León.

Species identification using Species Finder confirmed all isolates as *P. aeruginosa*. PFGE revealed distinct restriction patterns for each isolate, indicating genetic diversity among the strains (Figure 1). Accordingly, four pulsotypes were assigned: Psa/12-PT1, Psa/19-PT2, Psa/20-PT3, and Psa/21-PT4. Multilocus sequence typing (MLST), combined with *silico* serotyping via PAst, demonstrated that Psa/12, Psa/19, and Psa/21 belonged to ST309 and serotype O11, whereas Psa/20 was assigned to ST233 and serotype O6.

Genome assembly analysis showed that the ST233 isolate Psa/20 had the smallest genome (6,829,368 bp) among the analyzed isolates, whereas the ST309 isolates had larger genome sizes (Supplementary Table 1). Pairwise ANI comparisons corroborated the genomic relationships inferred by MLST (see Table 2). Nucleotide identity levels for the three ST309/O11 isolates ranged from 99.30% to 99.97%, with Psa/19 and Psa/21 showing nearly identical genomes (99.97%). In contrast to ST309 isolates, the ST233/O6 isolate Psa/20 exhibited lower average ANI values (98.7%–98.8 %) when compared to the ST309 isolates, which further supports the distinct ST classification of the ST233/O6 isolate (Table 2). Closely related ST309 isolates were identified in the analyzed hospitals, whereas isolates belonging to different STs exhibited greater genomic diversity.

**TABLE 2:** Pairwise ANI values of *Pseudomonas aeruginosa* clinical isolates.

Isolate	ANI (%)			
	Psa/12	Psa/19	Psa/20	Psa/21
Psa/12	100	99.95	98.72	99.30
Psa/19	99.95	100	98.79	99.97
Psa/20	98.72	98.79	100	98.78
Psa/21	99.30	99.97	98.78	100

Pairwise average nucleotide identity (ANI) values were calculated based on whole-genome comparisons and are expressed as percentage nucleotide identity.

### Resistome of *P. aeruginosa* high-risk clones

**FIGURE 2:**
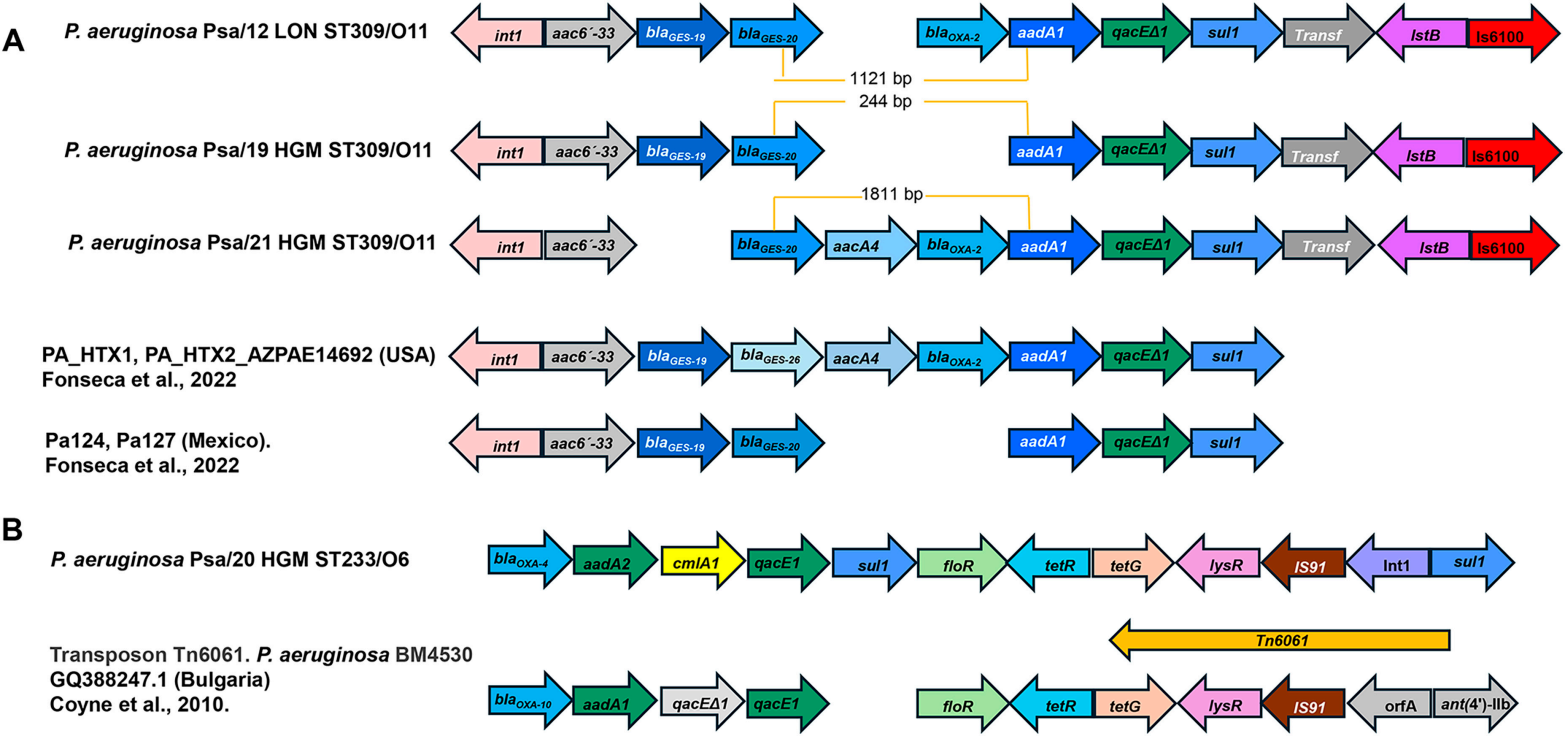
Genetic organization of variable resistance regions in *Pseudomonas aeruginosa* clinical isolates. **(A)** Structure of the integron-associated resistance region in ST309/O11 isolates (Psa/12, Psa/19, and Psa/21) compared with the model proposed by Fonseca et al. (2022). **(B)** Structure of the Tn6061 associated-resistance region in the ST233/O6 isolate Psa/20 compared with the transposon described in *P. aeruginosa* BM4530, modified from Coyne et al. (2010).

Whole-genome analysis provided evidence of specific ARGs content between the four isolates of *P. aeruginosa* derived from clinical sources. In all isolates, genes conferring resistance to β-lactams were predominant, including members of the *bla*_GES_, *bla*_PDC_, and *bla*_OXA_ families. Among the ST309/O11 isolates Psa/12 and Psa/19, 11 ARGs were identified, specifically, *bla*_GES–19_, *bla*_GES–20_, *bla*_PDC–19a_, *bla*_OXA–1035_, and *bla*_OXA–2_ were detected, although *bla*_OXA–2_ was absent in isolate Psa/19. The other ST309/O11 isolate Psa/21 harbored 12 ARGs, including *bla*_GES–20_, *bla*_PDC–19a_, *bla*_OXA–50_, and *bla*_OXA–2_. Whereas the ST233/O6 isolate Psa/20 exhibited the highest ARGs content, with a total of 16 resistance genes (Table 2). In contrast to Psa/12 and Psa/19, which also belong to ST309, Psa/21 lacked the *bla*_GES–19_ and *bla*_OXA–1035_ genes (Figure 2 and Table 3).

**TABLE 3:** Distribution of antimicrobial resistance genes and associated resistance phenotypes in *Pseudomonas aeruginosa* ST309 and ST233 clinical isolates.

No. isolate	Psa/12	Psa/19	Psa/20	Psa/21	Resistance phenotype
Sequence type/serotype	309/O11	309/O11	233/O6	309/O11	
Total resistance genes	11 genes	11 genes	16 genes	12 genes	
Antibiotic resistance genes (ARGs)	*sul1*	*sul1*	*sul1 / sul1*	*sul1*	Sulphonamide
	*fosA*	*fosA*	*fosA*	*fosA*	Fosfomycin
	–	*crpP*	*crpP*	*crpP*	Ciprofloxacin
	–	–	*tet(G)*	–	Tetracycline
	–	–	*dfrB5*	–	Trimethoprim
	*catB7*	*catB7*	–	*catB7*	Chloramphenicol
	–	–	*cmlA1*	–	
	–	–	*catB7*	–	
	*aph(3′)-IIb*	*aph(3′)-IIb*	*aph(3′)-IIb*	*aph(3′)-IIb*	Aminoglycosides
	*aac(6′)-33*	*aac(6′)-33*	–	*aac(6′)-33*	
	*aadA1b*	*aadA1b*	–	*aadA1b*	
	–	–	–	*aac(6′)-Ib3*	
	–	–	*aac(6′)-Il*	–	
	–	–	*aac(3)-Id*	–	
	–	–	*aadA2*	–	
	*bla*_*GES–19*_	*bla*_*GES–19*_	–	–	β-lactams
	*bla*_*GES–20*_	*bla*_*GES–20*_	–	*bla*_*GES–20*_	
	*bla*_*PDC–19a*_	*bla*_*PDC–19a*_	–	*bla*_*PDC–19a*_	
	–	–	*bla*_*PDC–3*_	–	
	–	–	–	*bla*_*OXA–50*_	
	*bla*_*OXA–1035*_	*bla*_*OXA–1035*_	–	–	
	–	–	*bla*_*OXA–486*_	–	
	*bla*_*OXA–2*_	–	–	*bla*_*OXA–2*_	
	–	–	*bla*_*OXA–4*_	–	
	–	–	*bla*_*VIM–2*_	–	Carbapenems

Interestingly, only the PDR isolate Psa/20 had the carbapenemase gene *bla*_VIM–2_. In addition, Psa/20 carried ARGs associated with resistance to aminoglycosides, chloramphenicol, sulfonamides, fosfomycin, fluoroquinolones, tetracyclines, and trimethoprim (Table 3). In contrast, the ST309 isolates lacked carbapenemase genes despite exhibiting high resistance values to imipenem and meropenem (MIC >128 μg/mL) (Table 1). None of the four isolates contained plasmids, according to an *in silico* plasmid prediction analysis, suggesting that all the factors determining resistance are chromosomally encoded.

Additional analyses of the accessory genome revealed structural variation among the isolates. Mobile Element Finder identified differences in insertion sequence-associated regions between the ST309 isolates (Supplementary Table 2). Likewise, prophage analysis performed using PHASTER detected variation in the number and estimated size of prophage regions across isolates. Psa/12, Psa/19, and Psa/21 carried four, six, and five prophage regions, respectively, with estimated total lengths of 125.2, 204.1, and 186 kb. In contrast, the ST233 isolate Psa/20 carried three prophage regions with an estimated total length of 104.7 kb (Supplementary Table 3).

**FIGURE 3:**
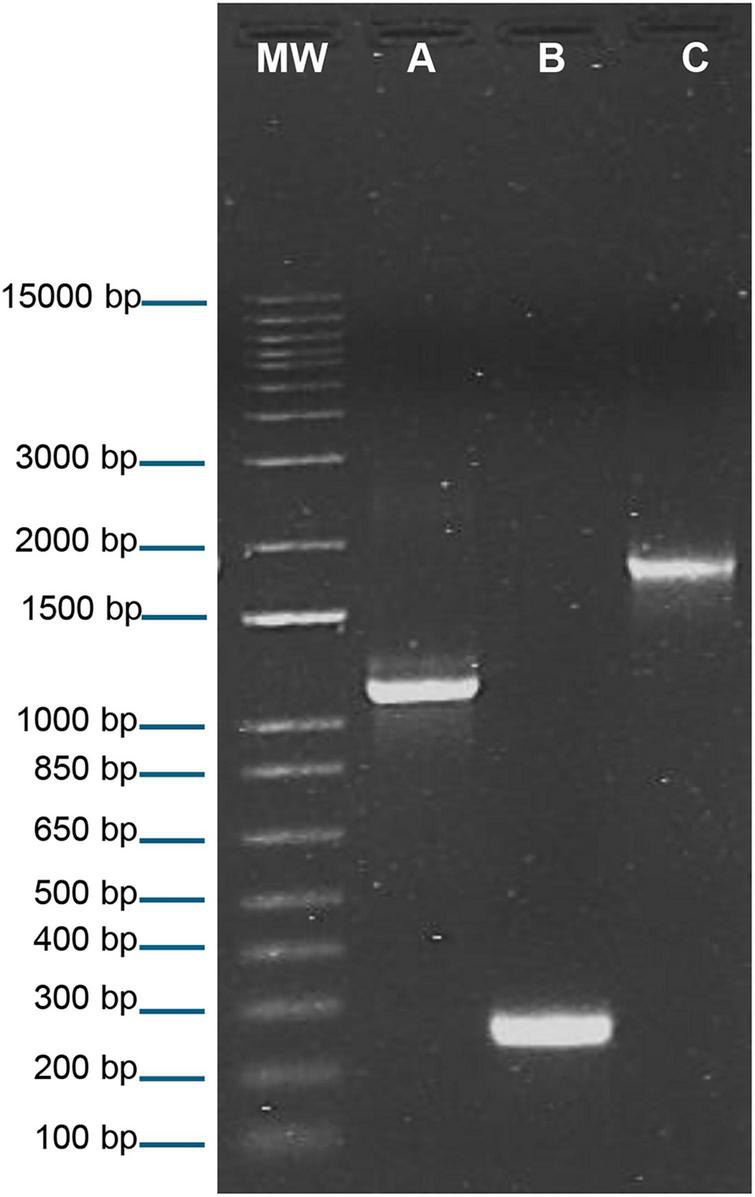
PCR amplification of variable resistance regions in *Pseudomonas aeruginosa* clinical isolates. Lane A: Psa/12 (LON, ST309/O11), 1,121 bp; Lane B: Psa/19 (HGM, ST309/O11), 244 bp; Lane C: Psa/21 (HGM, ST309/O11), 1,811 bp. MW: 1 kb Plus DNA ladder (molecular weight marker).

In addition to the overall resistome composition, a comprehensive analysis of the chromosomal resistance genes showed that the three ST309 isolates (Psa/12, Psa/19, and Psa/21) contained a conserved integron-associated region with five or six ARGs, flanked by intI1 and IS6100. This region contained genes (*aa6′-33, bla*_GES19_*, bla*_GES2_*_0_, aacA4, bla*_OXA–2_*, aadA1, qacEΔ1, sul1*) that encoded resistance to aminoglycosides, β-lactams, disinfectants, and sulfonamides (Figure 2). The ST309 isolates showed some structural variations, although the region has conserved its general structure and orientation. Psa/12 lacked the *aacA4* gene, Psa/19 lacked both *aacA4* and *bla*_OXA–2_, whereas Psa/21 lacked *bla*_GES–19_. These structural variations were experimentally confirmed by endpoint PCR using primers flanking the absent genes, yielding amplicons of 1121 bp for Psa/12, 244 bp for Psa/19, and 1811 bp for Psa/21 (Figures 2, 3).

Comparative genomic analysis with previously reported ST309 genomes demonstrated that the resistance cassette arrangements identified in Psa/19 were identical to those described for *P. aeruginosa* PA124 and PA127, isolated in Mexico in 2017 (Fonseca et al., 2022). Likewise, the arrangement observed in Psa/12 was comparable to that of isolate AZPAE14692 from the United States, except for the absence of *aacA4* in Psa/12. A similar comparison between Psa/21 and AZPAE14692 revealed the absence of *bla*_GES–19_ in Psa/21 (Figure 2). Gene sequence analysis in Psa/12 and Psa/19 revealed polymorphisms among *bla*_GES_ alleles, such as duplicated *bla*_GES–19_ and *bla*_GES–20_ alleles that differed by the G165S and A237G substitutions, and a non-synonymous substitution (G165S) that distinguished *bla*_GES–26_ from *bla*_GES–20_. The ST233 isolate Psa/20 showed a unique chromosomal multi-drug resistance region that was highly similar to the transposon Tn6061 and included the genes *tetG*, *lysR*, *IS91*, *int1*, and *sul1*, in contrast to the integron-based architecture shown in ST309 isolates. Interestingly, the Psa/20 isolate had two copies of the *sul1* gene. Of note, there is a substantial level of conservation in genomic architecture compared against the reference strain, *P. aeruginosa* BM4530 (GenBank: GQ388247), collected from Bulgaria in 2010 (Coyne et al., 2010; Figure 2). While the allelic variants of the *bla*_OXA_ and *aad* genes differed, Psa/20 and BM4530 shared the genes *qacE1*, *floR*, *tetR*, *tetG*, *lysR*, and *IS9*1.

Analysis of intrinsic resistance mechanisms revealed a wide range of efflux pump-associated genes in all isolates, in addition to acquired resistance determinants. The PDR isolate Psa/20 carried 48 efflux-related genes, whereas the remaining isolates harbored 47 genes each (Supplementary Table 4). The detected efflux systems belonged primarily to the Mex, Mux, Opm, and Opr families, which are associated with resistance to macrolides, fluoroquinolones, and phenolic compounds, as well as Tri-family pumps involved in antiseptic resistance.

To further explore resistance to last-resort antibiotics, sequence analysis of colistin resistance–associated regulatory systems revealed multiple amino acid substitutions compared with the colistin-susceptible reference strain PAO1. No amino acid substitutions were identified in PhoP in any of the analyzed isolates. In PhoQ, all four isolates exhibited a single substitution at the first residue (M1V). Mutations in the PmrAB regulatory system have previously been associated with lipid A modification and colistin resistance in *P. aeruginosa* (Lee and Ko, 2014). Consistent with these observations, the L71R substitution in PmrA was detected in the colistin-resistant isolates Psa/12, Psa/19, and Psa/21. In contrast, a distinct substitution (T31I) was identified in isolate Psa/20. Regarding PmrB, the colistin-resistant ST309 isolates (Psa/12, Psa/19, and Psa/21) carried the V15I and G68S substitutions, previously associated with intermediate colistin susceptibility. Additional substitutions linked to colistin resistance phenotypes, including S2P and A4T, were also detected in these isolates. The Y345H substitution was shared by all four isolates, while a unique substitution (A462T) was identified in Psa/20. No amino acid substitutions were detected in OprH. In contrast, NfeD exhibited multiple substitutions, including D114E, A169V, and S209G, which were present in all isolates. The L368V substitution was restricted to ST309 isolates (Psa/12, Psa/19, and Psa/21), whereas A146V and L403Q were unique to Psa/20.

**FIGURE 4:**
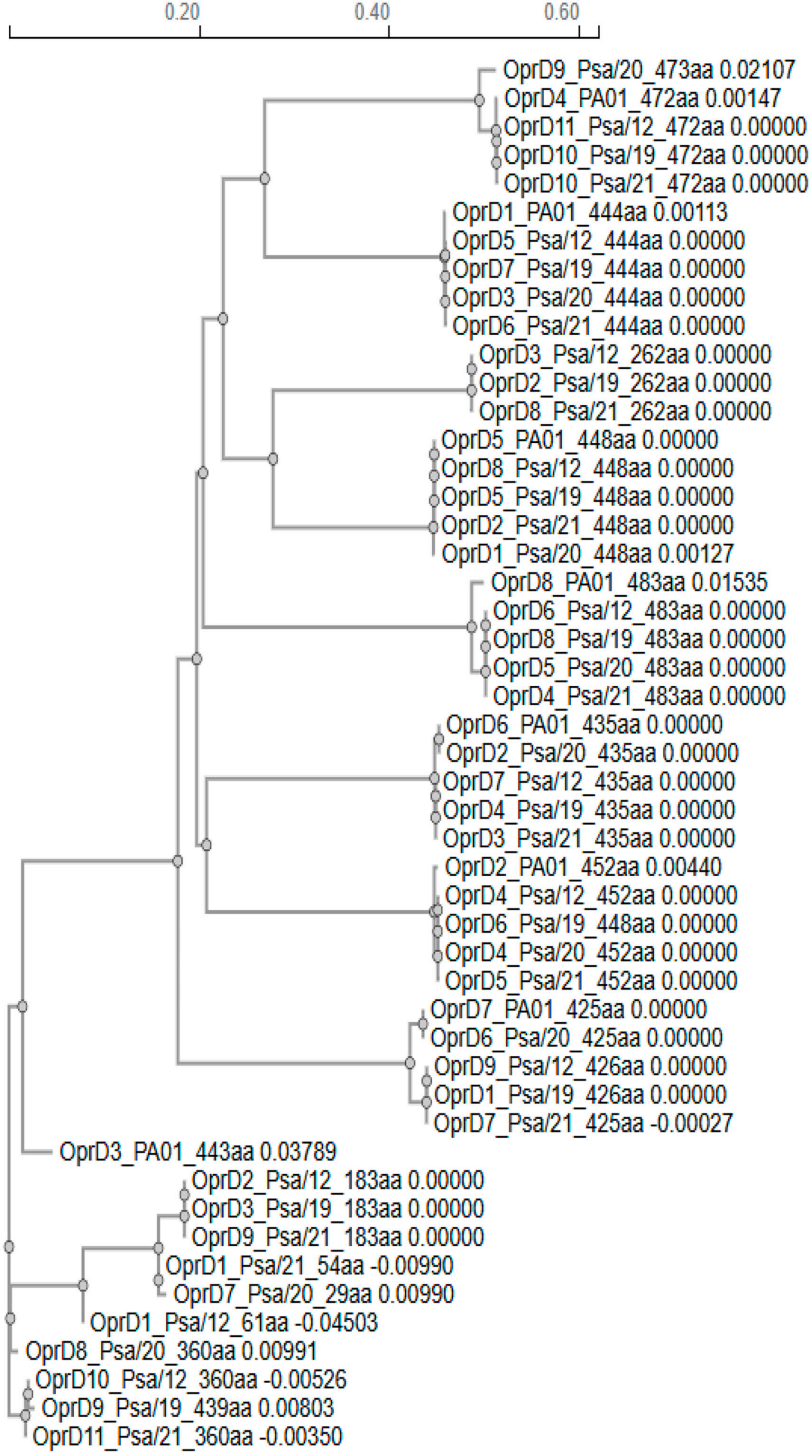
Distance matrix of OprD protein variants in *Pseudomonas aeruginosa* clinical isolates. Distance matrix constructed using the neighbor-joining algorithm for the OprD protein sequences identified in *P. aeruginosa* and the reference strain PAO1. OprD1–OprD11 represent the identified OprD allelic variants. Strains designated as Psa/ correspond to clinical isolates. “aa” indicates the amino acid length of the predicted open reading frame (ORF) of OprD.

We investigated additional resistance mechanisms, focusing on the OprD repertoire, because ST309 isolates exhibited carbapenem resistance despite lacking acquired carbapenemase genes. There were several open reading frames (ORFs) with homology to OprD found in Psa/12 (11), Psa/19 (10), Psa/20 (9), Psa/21 (11), and the reference strain PAO1 (8). The length of the putative OprD-related ORFs varied from 29 to 488 amino acids. Phylogenetic analysis of OprD homologs revealed two major branches diverging from the canonical OprD3_PAO1 protein (443 amino acids; ID: NP_249649.1) (Figure 4).

In the upper branches, several large OprD homologs showed 100% identity between PAO1 and the four clinical isolates, including OprD6_PA01 (435_amino acids). High sequence identity was also observed for OprD1_PA01 (444_amino acids), OprD8_PA01 (483_amino acids), OprD2_PA01 (452_amino acids), and OprD7_PA01 (425_amino acids). In the lower branches, shorter ORFs (29, 54, and 61 amino acids) were identified. Additional homologs, including OprD8_Psa/20 (360_amino acids) and OprD11_Psa/21 (360_amino acids), exhibited truncated variants lacking the N-terminal region. None of the OprD homologs identified in the clinical isolates were fully identical to the canonical 443-amino-acid OprD protein of PAO1.

### Virulome composition and differences between ST309 and ST233 isolates

Then, we examined the repertoire of virulence-associated genes of the isolates. The virulome analysis revealed a highly conserved virulence gene repertoire among the four *P. aeruginosa* isolates, with a comparable number of virulence-associated genes detected in each strain: 203 genes in Psa/12, 207 in Psa/19, 208 in Psa/20, and 209 in Psa/21 (Table 4). A core set of 199 virulence genes was shared by all isolates, regardless of ST, encompassing functions related to type IV pili-mediated twitching motility, alginate and rhamnolipid biosynthesis, hemolytic and non-hemolytic phospholipase C, siderophore production and uptake (pyochelin and pyoverdine systems), extracellular proteases (alkaline protease, elastase, and type IV protease), quorum sensing systems [N-(3-oxo-dodecanoyl)-L-homoserine lactone and N-(butanoyl)-L-homoserine lactone], the GacS/GacA two-component regulatory system, type III secretion system (T3SS), type VI secretion system (T6SS), exotoxin A (ETA), and hydrogen cyanide production (Table 4 and Supplementary Table 5).

**TABLE 4:** Comparative distribution of virulence factor classes and virulence genes among *Pseudomonas aeruginosa* ST 309 and ST233 clinical isolates.

Virulence factor class	Virulence factors	Strain	Psa/12	Psa/19	Psa/20	Psa/21
		Sequence type/serotype	ST309/O11	ST309/O11	ST233	ST309/O11
		Total virulence genes	203 genes	207 genes	208 genes	209 genes
		Virulence genes differentially present				
Adherence	Flagella	*flaG*	–	*flaG*	*flaG*	*flaG*
	Type IV pili biosynthesis	*fimT*	–	–	*fimT*	–
		*pilA*	–	*pilA*	*pilA*	*pilA*
Antimicrobial activity	Phenazines biosynthesis	*phzA1*	*phzA1*	*phzA1*	–	*phzA1*
		*phzA2*	–	–	–	*phzA2*
		*phzB2*	–	–	–	*phzB2*
Antiphagocytosis	Alginate regulation	*algP*	–	–	*algP*	*algP*
Enzyme	Phospholipase D	*pldA*	*pldA*	*pldA*	–	*pldA*
Iron uptake	Pyoverdine	*pvdD*	*pvdD*	*pvdD*	–	*pvdD*
		*pvdI*	–	*pvdI*	*pvdI*	–
		*pvdJ*	–	*pvdJ*	*pvdJ*	*pvdJ*
Secretion system	*P. aeruginosa* TTSS translocated effectors	*exoS*	–	–	*exoS*	–
		*exoU*	*exoU*	*exoU*	–	*exoU*
		*exoY*	–	–	*exoY*	–
	T6SS (*Aeromonas*)	*hcp1*	–	–	*hcp1*	–

Despite this extensive conservation, differences were identified in 14 virulence genes, revealing distinct virulence-associated profiles between ST309 and ST233 isolates (Table 4). Genes involved in phenazine biosynthesis (*phzA1*, *phzA2* and *phzB2*), phospholipase D (*pldA*), pyoverdine biosynthesis (*pvdD*), and the T3SS effector gene *exoU* were detected in at least one ST309 isolate (Psa/12, Psa/19, or Psa/21) but were absent in the ST233 isolate Psa/20. In contrast, genes encoding type IV pili-associated protein *fimT*, the T6SS component *hcp1*, and the T3SS effector genes *exoS* and *exoY* were exclusively identified in Psa/20 (ST233) (Table 4).

Analysis of adhesion and motility-associated determinants revealed variation in genes flagellar and pili-related genes (Table 4). The flagellar gene *flaG* was absent in isolate Psa/12, while the type IV pili-associated gene *fimT* was detected exclusively in Psa/20. The *pilA* gene, encoding the major pilin subunit of type IV pili, was absent in Psa/12. In total, 21 of the 26 *pil* genes described for *P. aeruginosa* were found in all isolates. In terms of fimbrial genes, only the ST233 isolate Psa/20 included *fimT*, while three of the five recognized *fim* genes were found.

Variability was also evident in phenazine biosynthesis pathways: the *phzA, phzA2 and phzB2* genes were absent in Psa/20 (Table 4), and *phzG1* was not detected in any of the four isolates; both these genes are involved in intermediate steps of the conversion of chorismic acid to pyocyanin. Regarding the *phzG2* gene, it was present in all strains. *phzA2* and *phzB2* were found exclusively in Psa/21 within the *phz* operon. All isolates had genes encoding the *phzM*, *phzS*, and *phzH* enzymes that modify phenazine (Supplementary Table 5).

All isolates, however, maintained the set of genes necessary for alginate biosynthesis, including the *algD* promoter region. However, the regulatory gene *algP* was absent in isolates Psa/12 and Psa/19, while all other alginate-associated regulatory genes were present (Supplementary Table 5).

Examination of phospholipase and siderophore-associated genes revealed differences among the isolates. The gene *pldA*, which is an effector of phospholipase D, was identified in all ST309 isolates (Psa/12, Psa/19, and Psa/21), but was absent in the ST233 isolate Psa/20. Regarding siderophore biosynthesis, the pyoverdine-associated gene *pvdD* (Ackerley and Lamont, 2004) was present in all ST309 isolates but absent in Psa/20. Two of the resistance genes, *pvdI* and *pvdJ*, were not confined to any particular ST and were observed to vary greatly across isolates.

Evidence was further provided supporting the virulence architecture through the differential distribution of effector proteins of the bacterial secretion system. All isolates harbored genes encoding structural components of the T3SS and T6SS. The distribution of T3SS effector genes differed between the analyzed ST309 and ST233 isolates: ST309 isolates carried *exoU* and lacked *exoS*, whereas Psa/20 (ST233) harbored *exoS* and lacked *exoU*. It’s interesting to note that all four isolates have *exoT*, but only Psa/20 had the effector gene *exoY*. With respect to the T6SS, all strains carried *hcp1* along with other conserved components of the HSI-I locus, including *clpV1*, *fha1*, *icmF1*, *ppkA*, *pppA*, and *vgrG1* (Supplementary Table 5).

## Discussion

The capacity of *P. aeruginosa* HRCs to survive prolonged antibiotic pressure, mediated by a variety of genetic and adaptive mechanisms, is increasingly linked to severe healthcare-associated infections (Torres-Rodríguez et al., 2026). Furthermore, genomic surveillance studies have demonstrated that these clones are capable of causing multidrug-resistant (MDR) outbreaks worldwide, thus highlighting their epidemiological impact (Aguilar-Rodea et al., 2017; Papa-Ezdra et al., 2024).

In our study, three of the four selected isolates exhibited an XDR phenotype (ST309/O11), while one isolate (ST233/O6) showed a PDR profile. Studies conducted in Mexican hospitals report XDR frequencies between 18% and 72%, as well as the presence of PDR isolates (Aguilar-Rodea et al., 2017; González-Olvera et al., 2019; Morales-Espinosa et al., 2017). These isolates showed reduced susceptibility to polymyxins and high resistance to carbapenems and fluoroquinolones, underscoring the decreasing availability of last-line treatment options for these clones.

Although PFGE revealed distinct pulsotypes among the analyzed isolates, whole-genome comparisons demonstrated high ANI values among the ST309 isolates, supporting strong conservation of the core genome within this ST. The differences observed in PFGE profiles may therefore reflect structural variability within the accessory genome, including integron-associated regions, insertion sequences, and prophages, which are known to influence restriction patterns. As a matter of fact, we found that the isolates have a different number of prophages.

The relationship between the isolates and their STs was further supported by serotype analysis. The PDR ST233 isolate corresponded to O6, while the ST309 isolates belonged to serotype O11, which is commonly linked to MDR/XDR clinical strains. Our results show that O11-associated ST309 isolates may exhibit XDR phenotypes within high-risk clonal backgrounds, although O11 and O12 serotypes have been associated with MDR phenotypes (Thrane et al., 2016). Both ST233 and ST309 are recognized as globally disseminated clones associated with outbreaks, resistance to last-line antibiotics, and poor clinical outcomes (Del Barrio-Tofiño et al., 2020; Papa-Ezdra et al., 2024; Khan et al., 2019; Hong et al., 2015; Abdouchakour et al., 2015).

The identification of the ST233 in an isolate from our study, which caused bacteremia in a patient at a tertiary care hospital and exhibited resistance to colistin and carbapenems, is consistent with previous findings from Mexico (Aguilar-Rodea et al., 2017) and other countries (Mudau et al., 2013), demonstrating the presence of this HRC in hospital settings. These findings support the importance of evaluating resistance phenotypes in their clonal context, since the differences in genomic composition may contribute to the variability of resistance-associated determinants among STs. The isolates analyzed in this study carried diverse chromosomal resistance determinants, including extended-spectrum β-lactamases, carbapenemases, and 16S rRNA methyltransferases (Lebreton et al., 2021), which are associated with MDR, XDR, and PDR phenotypes in *P. aeruginosa*.

The ST309/O11 isolates harbored 11-12 ARGs, predominantly β-lactam resistant genes (*bla*_GES_, *bla*_PDC_ and *bla*_OXA),_ organized within a conserved class 1 integron-associated region flanked by intI1 and IS6100. Although the overall cassette architecture was conserved, consistent structural differences among isolates, including the presence or absence of specific resistance genes, were experimentally confirmed by PCR. Comparative genomics analysis showed that these integron platforms closely resemble those reported in ST309 isolates from Mexico (Fonseca et al., 2022) and the United States (Khan et al., 2019), indicating conservation of related chromosomal resistance regions among geographically distinct ST309 isolates.

Cassette rearrangements, gene duplication events, and non-synonymous mutations (e.g., G165S distinguishing *bla*_GES–26_ from *bla*_GES–20_) may contribute to resistance mechanisms in *P. aeruginosa* under antibiotic selective pressure. In contrast, the PDR isolate Psa/20 exhibited a distinct resistance architecture that included the carbapenemase *bla*_VIM–2_ and a chromosomal MDR region highly syntenic with the Tn6061-like structure previously reported in strain BM4530 from Europe more than a decade ago (Coyne et al., 2010). Previous reports of this genetic arrangement may suggest that certain resistance regions can remain conserved over extended periods of time. Notably, all resistance determinants identified in our isolates were detected *in silico* within chromosomal regions, while no plasmid-associated resistance elements were identified.

Additional analyses of accessory genomic elements revealed structural variability among the analyzed isolates. Mobile Element Finder identified differences in insertion sequence-associated regions between the ST309 isolates (Supplementary Table 2). Likewise, prophage analysis performed using PHASTER detected variation in the number and estimated size of prophage regions across isolates. Psa/12, Psa/19, and Psa/21 carried four, six, and five prophage regions, respectively, whereas the ST233 isolate Psa/20 carried three prophage regions (Supplementary Table 3).

In addition to acquired ARGs, all isolates carried multiple intrinsic resistance-associated factors, including efflux systems belonging to the Mex, Mux, Opm, Opr, and Tri families. Similar efflux-related gene content was identified among XDR and PDR isolates.

Notably, diversification and truncation of OprD homologs have previously been associated with carbapenem resistance in ST309 isolates lacking acquired carbapenemase genes (González-Vázquez et al., 2021). Multiple OprD-related ORFs were identified in our isolates (Figure 4), consistent with previous reports describing OprD as the prototype of a large paralogous porin family in *P. aeruginosa*, comprising at least 18 homologs sharing 46%–57% amino acid similarity (Tamber et al., 2006; Chevalier et al., 2017). This genomic diversity may contribute to structural and functional variability within the OprD family. Additionally, compared with PAO1, none of the clinical isolates retained a fully conserved OprD protein, suggesting that porin alterations could contribute to the carbapenem resistance phenotype observed in these isolates, potentially in combination with other resistance mechanisms. Nevertheless, the involvement of additional resistance mechanisms, including efflux pump systems and regulatory pathways, cannot be ruled out. Regarding colistin resistance in the PDR ST233 isolate, our findings identified mutations in two-component regulatory systems involved in lipid A modification (PhoPQ, PmrAB), as well as novel and previously described *nfeD* (Alarcon Rios et al., 2025; Zarate et al., 2021). The amino acid substitutions identified in NfeD suggest potential variability in this protein among high-risk *P. aeruginosa* clones. Notably, several substitutions (D114E, A169V, and S209G) were conserved across all analyzed isolates, while additional variants showed sequence type-specific distribution, with L368V restricted to ST309 isolates and A146V and L403Q unique to the ST233 isolate. Importantly, the variants identified in this study differ from those reported by Alarcon Rios et al. (2025), who described a synonymous mutation in an *in vitro* adaptation model. These differences likely reflect distinct selective environments, as clinical isolates are subject to complex and prolonged antimicrobial pressure *in vivo*, whereas *in vitro* models represent controlled and directional selection conditions. However, the contribution of NfeD substitutions to colistin resistance or broader adaptive fitness in *P. aeruginosa* remains to be experimentally validated. These findings indicate that the resistance profiles observed in these isolates are associated with multiple chromosomal resistance-associated mechanisms, including integron-associated resistance regions, OprD alterations, efflux-related determinants, and regulatory systems mutations.

A combination of antimicrobial resistance and virulence factors makes it possible for *P. aeruginosa* to persist in the healthcare setting (Veetilvalappil et al., 2022). We conducted a virulome analysis of our isolates, which revealed that they share a similar core structure with 199 common genes that related to type-IV pili-mediated motility; alginate and biofilm formation; rhamnolipid production; phospholipase activity, quorum-sensing, acquisition of iron by means of siderophore(s), extracellular proteolysis, and type-III and –VI secretion systems, all of which are commonly described in healthcare-associated *P. aeruginosa* isolates as virulence determinants.

Despite this shared backbone, 14 virulence-associated genes exhibited a genome-specific distribution, supporting a variable virulence architecture. Adhesion-related variability was particularly evident in type IV pili-associated genes. *P. aeruginosa* possesses 26 *pil* genes (*pilA*–*pilZ*) and five *fim* genes involved in type IV pilus formation and function (Veetilvalappil et al., 2022). Among the *fim* genes evaluated, *fimT* was detected only in the PDR strain Psa/20. Previous studies demonstrated that FimT proteins can interact with extracellular DNA and contribute to natural transformation processes in Gram-negative bacteria (Braus et al., 2022). Therefore, the presence of *fimT* in Psa/20 may suggest a potential role in extracellular DNA uptake mechanisms. Interestingly, this isolate also harbored the highest number of resistance genes, including *bla*_OXA–486_ and *bla*_VIM–2_. However, additional functional studies are required to determine whether *fimT* contributes to DNA acquisition and resistance gene incorporation in these isolates.

Regarding phenazine biosynthesis, the four isolates exhibited partially overlapping gene distributions. Our findings showed that none of the isolates carried both complete operons, as *phzG1* was absent in all strains. Furthermore, isolate ST233 lacked *phzA1*, *phzA2*, and *phzB2*. The fact that all isolates had the modifying enzymes (*phzM*, *phzS*, and *phzH*) and *phzG2* indicates that they have similar functions (Yu et al., 2025), which have been linked to redox homeostasis and stress resistance during the formation of biofilms in *P. aeruginosa* (Vilaplana and Marco, 2020).

The composition of genes responsible for alginate biosynthesis was mostly conserved, including the *algD* promoter region, and only the Psa/12 and Psa/19 isolates lacked *algP*. Since alginate production may still depend on c-di-GMP signaling (Cross et al., 2020; Liang et al., 2022), these profiles remain consistent with the biofilm capacity maintained in all isolated strains, reinforcing the link between biofilm-associated virulence traits and antibiotic tolerance.

Genome-specific differences were also evident in host interaction and interbacterial competition. *pldA*, an effector of phospholipase D (Yang et al., 2022), was present in only one ST309 isolate; these variations could affect niche adaptation in polymicrobial environments, such as those found in hospitals. Iron acquisition is crucial for the pathogenicity of *P. aeruginosa*. In our research, the siderophore-associated genes (*pvdD*, *pvdI*, and *pvdJ*) exhibited heterogeneous distribution across isolates, indicating that siderophore production does not depend strictly on ST (Bonneau et al., 2020). Additionally, T3SS effector profiles adhered to the conventional exoU/exoS dichotomy: ST233 isolates had *exoS* and *exoY* but lacked *exoU*, suggesting an invasive profile, whereas ST309 isolates carried *exoU* and lacked *exoS*, consistent with a cytotoxic phenotype (Akter et al., 2025).

Our study has some limitations. The main one is the limited sample size considered. Another limitation is that the isolates come from just two hospitals. Nonetheless, our findings suggest interesting trends that should be validated in larger collections of isolates. Future studies should further explore these observations to better understand their epidemiological and clinical significance of these high-risk clones.

Taken together, the results demonstrate that high-risk *P. aeruginosa* clones circulating in Mexican hospitals share a conserved repertoires of virulence-associated genes together with diverse ARGs. The conservation of these genomic features in healthcare settings, where challenges to the ability to survive and persist are reported, likely enhances the tolerance of *P. aeruginosa* to various antimicrobial agents while simultaneously promoting persistence in healthcare settings.

Beyond confirming the circulation of globally disseminated high-risk clones, this study provides an integrated genomic comparison of ST309 and ST233 isolates recovered from Mexican hospitals, combining analyses of integron architecture, chromosomal resistance determinants, accessory genomic elements, and virulence-associated gene content. These observations contribute to a better understanding of the genomic features that may facilitate the persistence and adaptation of high-risk *P. aeruginosa* clones in healthcare environments.

## Conclusion

This study documents the presence of high-risk *P. aeruginosa* clones previously reported in Mexican hospitals, including ST309 and ST233 isolates exhibiting XDR phenotypes. Among the ST309 isolates analyzed, a largely conserved repertoire of resistance genes was identified, although genetic variability was observed within integron-associated resistance regions, with structural differences detected among closely related isolates. Virulence-associated genes were broadly conserved among the analyzed isolates; however, differences were observed between ST309 and ST233, including variation in the distribution of ExoS and ExoU associated repertoires. Additional ST233 isolates will be required to further support these observations. Furthermore, although multiple OprD-related ORFs were identified in all isolates, none retained the conserved structure observed in the PAO1 reference strain, consistent with previous reports associating OprD alterations with carbapenem resistance. Overall, these findings provide a genomic characterization of high-risk *P. aeruginosa* isolates recovered from Mexican hospitals and support the importance of continued genomic surveillance to monitor the evolution and dissemination of clinically relevant resistance and virulence-associated determinants.

## Data Availability

The datasets presented in this study can be found in online repositories. The names of the repository/repositories and accession number(s) can be found in the article/Supplementary material.
