# Supplementary material for: Integron architecture and virulence-associated gene profiles in extensively drug-resistant high-risk Pseudomonas aeruginosa ST309 and ST233
Source: Front Microbiol. 2026 Jul 23;17:1833035. doi: 10.3389/fmicb.2026.1833035 (PMC13442380; doi:10.3389/fmicb.2026.1833035)
Supplement: Supplementary file 1 [file Table_1.docx]

**Supplementary table 1. Genome assembly statistics in *Pseudomonas aeruginosa* ST309 and ST233 clinical isolates*.***

|  | **N50** | **Contigs**  **number** | **Total assembly length (bp)** | **Sequencing Coverage** |
| --- | --- | --- | --- | --- |
| **Psa/12** | 342256 | 281 | 6937963 | 112 X |
| **Psa/19** | 359089 | 245 | 7007789 | 130 X |
| **Psa/20** | 442785 | 139 | 6829368 | 99 X |
| **Psa/21** | 305150 | 1209 | 7513238 | 89 X |
