# Supplementary material for: Integron architecture and virulence-associated gene profiles in extensively drug-resistant high-risk Pseudomonas aeruginosa ST309 and ST233
Source: Front Microbiol. 2026 Jul 23;17:1833035. doi: 10.3389/fmicb.2026.1833035 (PMC13442380; doi:10.3389/fmicb.2026.1833035)
Supplement: Supplementary file 2 [file Table_2.docx]

**Supplementary table 2. Insertion sequences (IS) identified in *Pseudomonas aeruginosa* ST309 and ST233 clinical isolates*.***

| **Isolate** |  | **Psa/12** | **Psa/19** | **Psa/21** | **Isolate** |  | **Psa/20** |
| --- | --- | --- | --- | --- | --- | --- | --- |
| **Sequence Type /Serotype** |  | **ST309/O11** | **ST309/O11** | **ST309/O11** | **Sequence Type /Serotype** |  | **ST233/O6** |
| **Insertion Type** | **IS type** | **Allele length bp** | **Allele length bp** | **Allele length bp** | **Insertion Type** | **IS type** | **Allele length bp** |
| Insertion sequence | ISPa6 | 1333 | 1333 | 1333 | Unit transposon | Tn4661 | 12706 |
| Insertion sequence | ISPa1 | 1033 | 1033 | 1033 | Insertion sequence | ISPsy29 | 1231 |
| Insertion sequence | ISPa32 | 1243 | 1243 | 1243 | Insertion sequence | ISPa2 | 788 |
| Unit transposon | Tn5563 | 6253 | 6253 | 6253 | Unit transposon | Tn6061 | 4000 |
| Insertion sequence | ISPst12 | 1331 | 1331 | 1331 | Insertion sequence | IS6100 | 880 |
| Insertion sequence | ISPa32 | 1236 | 1236 | 1236 | Insertion sequence | ISPa1328 | 1328 |
| Insertion sequence | IS6100 | 880 | 880 | 880 | Total length |  | 20933 bp |
| Insertion sequence | ISPa1328 | 1328 | 1328 | 1328 |  |  |  |
| Composite transposon | cn_11516_ISPa32 |  |  | 11516 |  |  |  |
| Total length |  | 14637 bp | 14637 bp | 26153 bp |  |  |  |
