# Supplementary material for: Integron architecture and virulence-associated gene profiles in extensively drug-resistant high-risk Pseudomonas aeruginosa ST309 and ST233
Source: Front Microbiol. 2026 Jul 23;17:1833035. doi: 10.3389/fmicb.2026.1833035 (PMC13442380; doi:10.3389/fmicb.2026.1833035)
Supplement: Supplementary file 3 [file Table_3.docx]

**Supplementary Table 3. Prophage sequence identified in *Pseudomonas aeruginosa* ST309 and ST233 clinical isolates*.***

| **Isolate** | **Psa/12** | | **Isolate** | **Psa/19** | | **Isolate** | **Psa/21** | | **Isolate** | **Psa/20** | |
| --- | --- | --- | --- | --- | --- | --- | --- | --- | --- | --- | --- |
| **Sequence Type /Serotype** | **ST309/O11** | | **Sequence Type /Serotype** | **ST309/O11** | | **Sequence Type /Serotype** | **ST309/O11** | | **Sequence Type /Serotype** | **ST233/O6** | |
| **Prophage identified** | **Region length Kb** | **Total number proteins** | **Prophage identified** | **Region length Kb** | **Total number proteins** | **Prophage identified** | **Region length Kb** | **Total number proteins** | **Prophage identified** | **Region length Kb** | **Total number proteins** |
| PHAGE_Pseudo_MD8 | 41.4 Kb | 57 | PHAGE_Pseudo_Pf1 | 16.2Kb | 12 | PHAGE_Pseudo_Pf1 | 16.2Kb | 12 | PHAGE_Pseudo_H6 | 64.5Kb | 70 |
| PHAGE_Pseudo_Pf1 | 18.6 Kb | 12 | PHAGE_Pseudo_D3 | 57.8Kb | 84 | PHAGE_Pseudo_Dobby | 46.8Kb | 53 | PHAGE_Pseudo_YMC11/02/R656 | 30.6Kb | 38 |
| PHAGE_Pseudo_Doby | 46.8 Kb | 52 | PHAGE_Pseudo_Dobby | 46.8Kb | 52 | PHAGE_Pseudo_H6 | 64.9Kb | 70 | PHAGE_Pseudo_Dbby | 36.6Kb | 46 |
| PHAGE_Pseudo_Doby | 18.4 Kb | 25 | PHAGE_Pseudo_MD8 | 44.5Kb | 36 | PHAGE_Pseudo_vBPaeS | 39.7Kb | 55 | Total length | 104.7 kb |  |
| Total length | 125.2 Kb |  | PHAGE_Pseudo_phiCTX | 18.4Kb | 25 | PHAGE_Pseudo_phiCTX | 18.4Kb | 25 | Total proteins |  | 234 |
| Total proteins |  | 146 | PHAGE_Pseudo_vB_PaeS_PM105 | 20.4Kb | 25 | Total length | 186 Kb |  |  |  |  |
|  |  |  | Total length | 204.1 Kb |  | Total proteins |  | 234 |  |  |  |
|  |  |  | Total proteins |  | 234 |  |  |  |  |  |  |
