# Supplementary material for: Integron architecture and virulence-associated gene profiles in extensively drug-resistant high-risk Pseudomonas aeruginosa ST309 and ST233
Source: Front Microbiol. 2026 Jul 23;17:1833035. doi: 10.3389/fmicb.2026.1833035 (PMC13442380; doi:10.3389/fmicb.2026.1833035)
Supplement: Supplementary file 4 [file Table_4.docx]

**Supplementary Table 4. Efflux pump gene repertoire and associated resistance phenotypes in *Pseudomonas aeruginosa* ST309 and ST233 clinical isolates.**

|  | **Strain** | **Psa/12** | **Psa/19** | **Psa/20** | **Psa/21** |
| --- | --- | --- | --- | --- | --- |
|  | **Sequence type/ Serotype** | **309/O11** | **309/O11** | **233/O6** | **309/O11** |
|  | **Genes involved in efflux systems** | **Total efflux pump genes** | | | |
|  |  | **47 genes** | **47 genes** | **48 genes** | **47 genes** |
| **Resistance phenotype** |  | | | | |
| Fluoroquinolone | *adeF* | *adeF* |  |  |  |
| Antibiotic efflux | *armR* |  |  | *armR* |  |
| bicyclomycin-like antibiotic | *bcr-1* | *bcr-1* | *bcr-1* | *bcr-1* | *bcr-1* |
| Macrolide | *cpxR* | *cpxR* | *cpxR* | *cpxR* | *cpxR* |
| Aminoglycoside | *emrE* | *emrE* | *emrE* | *emrE* | *emrE* |
| Phenicol antibiotic | *cmlA6* |  |  | *cmlA6* |  |
| Phenicol antibiotic | *cmlA9* |  |  | *cmlA9* |  |
| Macrolide | *mexA* | *mexA* | *mexA* | *mexA* | *mexA* |
| Macrolide | *mexB* | *mexB* | *mexB* | *mexB* | *mexB* |
| Macrolide | *mexC* | *mexC* | *mexC* | *mexC* | *mexC* |
| Macrolide | *mexD* | *mexD* | *mexD* | *mexD* | *mexD* |
| Fluoroquinolone | *mexE* | *mexE* | *mexE* | *mexE* | *mexE* |
| Fluoroquinolone | *mexF* |  | *mexF* | *mexF* | *MexF* |
| Fluoroquinolone | *mexG* | *mexG* | *mexG* | *mexG* | *mexG* |
| Fluoroquinolone | *mexH* | *mexH* | *mexH* | *mexH* | *mexH* |
| Fluoroquinolone | *mexI* | *mexI* | *mexI* | *mexI* | *mexI* |
| Macrolide | *mexJ* | *mexJ* | *mexJ* | *mexJ* | *mexJ* |
| Macrolide | *mexK* | *mexK* | *mexK* | *mexK* | *mexK* |
| Phenicol antibiotic | *mexM* | *mexM* | *mexM* | *mexM* | *mexM* |
| Phenicol antibiotic | *mexN* | *mexN* | *mexN* | *mexN* | *mexN* |
| Macrolide | *mexP* | *mexP* | *mexP* | *mexP* | *mexP* |
| Macrolide | *mexQ* | *mexQ* | *mexQ* | *mexQ* | *mexQ* |
| Antibiotic efflux | *mexR* | *mexR* | *mexR* | *mexR* | *mexR* |
| Fluoroquinolone | *mexS* | *mexS* | *mexS* | *mexS* | *mexS* |
| Fluoroquinolone | *mexT* | *mexT* | *mexT* | *mexT* | *mexT* |
| Macrolide | *mexV* | *mexV* | *mexV* | *mexV* | *mexV* |
| Macrolide | *mexW* | *mexW* | *mexW* | *mexW* | *mexW* |
| Macrolide | *mexY* | *mexY* | *mexY* | *mexY* | *mexY* |
| Macrolide | *mexZ* | *mexZ* | *mexZ* | *mexZ* | *mexZ* |
| Macrolide | *muxA* | *muxA* | *muxA* |  | *muxA* |
| Macrolide | *muxB* | *muxB* | *muxB* | *muxB* | *muxB* |
| Macrolide | *muxC* | *muxC* | *muxC* | *muxC* | *muxC* |
| Antibiotic efflux | *nalC* | *nalC* | *nalC* | *nalC* | *nalC* |
| Antibiotic efflux | *nalD* | *nalD* | *nalD* | *nalD* | *nalD* |
| Macrolide | *opmB* | *opmB* | *opmB* | *OpmB* | *opmB* |
| Fluoroquinolone | *opmD* | *opmD* | *opmD* | *opmD* | *opmD* |
| Macrolide | *opmE* | *opmE* | *opmE* | *opmE* | *opmE* |
| Disinfecting agents and antiseptics | *opmH* | *opmH* | *opmH* | *opmH* | *opmH* |
| Macrolide | *oprJ* | *oprJ* | *oprJ* | *oprJ* | *oprJ* |
| Macrolide | *oprM* | *oprM* | *oprM* | *oprM* | *oprM* |
| Fluoroquinolone | *oprN* | *oprN* | *oprN* | *oprN* | *oprN* |
| Macrolide | *parR* | *parR* | *parR* | *parR* | *parR* |
| Macrolide | *parS* | *parS* | *parS* | *parS* | *parS* |
| Fluoroquinolone | *pmpM* | *pmpM* | *pmpM* | *pmpM* | *pmpM* |
| Disinfecting agents and antiseptics | *qacEdelta1* | *qacEdelta1* | *qacEdelta1* | *qacEdelta1* | *qacEdelta1* |
| Fluoroquinolone | *rsmA* | *rsmA* | *rsmA* | *rsmA* | *rsmA* |
| Disinfecting agents and antiseptics | *triA* | *triA* | *triA* |  | *triA* |
| Disinfecting agents and antiseptics | *triB* | *triB* | *triB* | *triB* | *triB* |
| Disinfecting agents and antiseptics | *triC* | *triC* | *triC* | *triC* | *triC* |
| Macrolide | *nfxB Type A* | *nfxB Type A* | *Type A nfxB* | *Type A nfxB* | *Type A nfxB* |
| Fluoroquinolone | *yajC* | *yajC* | *yajC* | *yajC* | *yajC* |
