# Supplementary material for: Integron architecture and virulence-associated gene profiles in extensively drug-resistant high-risk Pseudomonas aeruginosa ST309 and ST233
Source: Front Microbiol. 2026 Jul 23;17:1833035. doi: 10.3389/fmicb.2026.1833035 (PMC13442380; doi:10.3389/fmicb.2026.1833035)
Supplement: Supplementary file 5 [file Table_5.docx]

**Supplementary Table 5. Complete virulome profile of *Pseudomonas aeruginosa* ST309 and ST233 clinical isolates, including virulence classes, determinants, and associated genes.**

| **Virulome** | | |  |  |  |  |  |
| --- | --- | --- | --- | --- | --- | --- | --- |
| **Virulence factors class** | **Virulence factors** | **Gene** | **Psa/12** | **Psa/19** | **Psa/20** | **Psa/21** |  |
|  |  |  |  |  |  |  |  |
| Adherence | Flagella | *flaG* | *-* | *flaG* | *flaG* | *flaG* |  |
|  |  | *fleN* | *fleN* | *fleN* | *fleN* | *fleN* |  |
|  |  | *fleQ* | *fleQ* | *fleQ* | *fleQ* | *fleQ* |  |
|  |  | *fleR* | *fleR* | *fleR* | *fleR* | *fleR* |  |
|  |  | *fleS* | *fleS* | *fleS* | *fleS* | *fleS* |  |
|  |  | *flgA* | *flgA* | *flgA* | *flgA* | *flgA* |  |
|  |  | *flgB* | *flgB* | *flgB* | *flgB* | *flgB* |  |
|  |  | *flgC* | *flgC* | *flgC* | *flgC* | *flgC* |  |
|  |  | *flgD* | *flgD* | *flgD* | *flgD* | *flgD* |  |
|  |  | *flgE* | *flgE* | *flgE* | *flgE* | *flgE* |  |
|  |  | *flgF* | *flgF* | *flgF* | *flgF* | *flgF* |  |
|  |  | *flgG* | *flgG* | *flgG* | *flgG* | *flgG* |  |
|  |  | *flgH* | *flgH* | *flgH* | *flgH* | *flgH* |  |
|  |  | *flgI* | *flgI* | *flgI* | *flgI* | *flgI* |  |
|  |  | *flgJ* | *flgJ* | *flgJ* | *flgJ* | *flgJ* |  |
|  |  | *flgK* | *flgK* | *flgK* | *flgK* | *flgK* |  |
|  |  | *flgL* | *flgL* | *flgL* | *flgL* | *flgL* |  |
|  |  | *flgM* | *flgM* | *flgM* | *flgM* | *flgM* |  |
|  |  | *flgN* | *flgN* | *flgN* | *flgN* | *flgN* |  |
|  |  | *flhA* | *flhA* | *flhA* | *flhA* | *flhA* |  |
|  |  | *flhB* | *flhB* | *flhB* | *flhB* | *flhB* |  |
|  |  | *flhF* | *flhF* | *flhF* | *flhF* | *flhF* |  |
|  |  | *fliA* | *fliA* | *fliA* | *fliA* | *fliA* |  |
|  |  | *fliC* | *fliC* | *fliC* | *fliC* | *fliC* |  |
|  |  | *fliD* | *fliD* | *fliD* | *fliD* | *fliD* |  |
|  |  | *fliE* | *fliE* | *fliE* | *fliE* | *fliE* |  |
|  |  | *fliF* | *fliF* | *fliF* | *fliF* | *fliF* |  |
|  |  | *fliG* | *fliG* | *fliG* | *fliG* | *fliG* |  |
|  |  | *fliH* | *fliH* | *fliH* | *fliH* | *fliH* |  |
|  |  | *fliI* | *fliI* | *fliI* | *fliI* | *fliI* |  |
|  |  | *fliJ* | *fliJ* | *fliJ* | *fliJ* | *fliJ* |  |
|  |  | *fliK* | *fliK* | *fliK* | *fliK* | *fliK* |  |
|  |  | *fliL* | *fliL* | *fliL* | *fliL* | *fliL* |  |
|  |  | *fliM* | *fliM* | *fliM* | *fliM* | *fliM* |  |
|  |  | *fliN* | *fliN* | *fliN* | *fliN* | *fliN* |  |
|  |  | *fliO* | *fliO* | *fliO* | *fliO* | *fliO* |  |
|  |  | *fliP* | *fliP* | *fliP* | *fliP* | *fliP* |  |
|  |  | *fliQ* | *fliQ* | *fliQ* | *fliQ* | *fliQ* |  |
|  |  | *fliR* | *fliR* | *fliR* | *fliR* | *fliR* |  |
|  |  | *fliS* | *fliS* | *fliS* | *fliS* | *fliS* |  |
|  |  | *fliT* | *fliT* | *fliT* | *fliT* | *fliT* |  |
|  |  | *motA* | *motA* | *motA* | *motA* | *motA* |  |
|  |  | *motB* | *motB* | *motB* | *motB* | *motB* |  |
|  |  | *motC* | *motC* | *motC* | *motC* | *motC* |  |
|  |  | *motD* | *motD* | *motD* | *motD* | *motD* |  |
|  |  | *motY* | *motY* | *motY* | *motY* | *motY* |  |
|  | Type IV pili biosynthesis | *fimT* | *-* | *-* | *fimT* | *-* |  |
|  |  | *fimU* | *fimU* | *fimU* | *fimU* | *fimU* |  |
|  |  | *fimV* | *fimV* | *fimV* | *fimV* | *fimV* |  |
|  |  | *pilA* | *-* | *pilA* | *pilA* | *pilA* |  |
|  |  | *pilB* | *pilB* | *pilB* | *pilB* | *pilB* |  |
|  |  | *pilC* | *pilC* | *pilC* | *pilC* | *pilC* |  |
|  |  | *pilD* | *pilD* | *pilD* | *pilD* | *pilD* |  |
|  |  | *pilE* | *pilE* | *pilE* | *pilE* | *pilE* |  |
|  |  | *pilF* | *pilF* | *pilF* | *pilF* | *pilF* |  |
|  |  | *pilM* | *pilM* | *pilM* | *pilM* | *pilM* |  |
|  |  | *pilN* | *pilN* | *pilN* | *pilN* | *pilN* |  |
|  |  | *pilO* | *pilO* | *pilO* | *pilO* | *pilO* |  |
|  |  | *pilP* | *pilP* | *pilP* | *pilP* | *pilP* |  |
|  |  | *pilQ* | *pilQ* | *pilQ* | *pilQ* | *pilQ* |  |
|  |  | *pilR* | *pilR* | *pilR* | *pilR* | *pilR* |  |
|  |  | *pilS* | *pilS* | *pilS* | *pilS* | *pilS* |  |
|  |  | *pilT* | *pilT* | *pilT* | *pilT* | *pilT* |  |
|  |  | *pilU* | *pilU* | *pilU* | *pilU* | *pilU* |  |
|  |  | *pilV* | *pilV* | *pilV* | *pilV* | *pilV* |  |
|  |  | *pilW* | *pilW* | *pilW* | *pilW* | *pilW* |  |
|  |  | *pilX* | *pilX* | *pilX* | *pilX* | *pilX* |  |
|  |  | *pilY1* | *pilY1* | *pilY1* | *pilY1* | *pilY1* |  |
|  |  | *pilY2* | *pilY2* | *pilY2* | *pilY2* | *pilY2* |  |
|  |  | *pilZ* | *pilZ* | *pilZ* | *pilZ* | *pilZ* |  |
|  | Type IV pili twitching motility related proteins | *chpA* | *chpA* | *chpA* | *chpA* | *chpA* |  |
|  |  | *chpB* | *chpB* | *chpB* | *chpB* | *chpB* |  |
|  |  | *chpC* | *chpC* | *chpC* | *chpC* | *chpC* |  |
|  |  | *chpD* | *chpD* | *chpD* | *chpD* | *chpD* |  |
|  |  | *chpE* | *chpE* | *chpE* | *chpE* | *chpE* |  |
|  |  | *pilG* | *pilG* | *pilG* | *pilG* | *pilG* |  |
|  |  | *pilH* | *pilH* | *pilH* | *pilH* | *pilH* |  |
|  |  | *pilI* | *pilI* | *pilI* | *pilI* | *pilI* |  |
|  |  | *pilJ* | *pilJ* | *pilJ* | *pilJ* | *pilJ* |  |
|  |  | *pilK* | *pilK* | *pilK* | *pilK* | *pilK* |  |
| Antimicrobial activity | Phenazines biosynthesis | *phzA1* | *phzA1* | *phzA1* | *-* | *phzA1* |  |
|  |  | *phzA2* | *-* | *-* | *-* | *phzA2* |  |
|  |  | *phzB1* | *phzB1* | *phzB1* | *phzB1* | *phzB1* |  |
|  |  | *phzB2* | *-* | *-* | *-* | *phzB2* |  |
|  |  | *phzC1* | *phzC1* | *phzC1* | *phzC1* | *phzC1* |  |
|  |  | *phzD1* | *phzD1* | *phzD1* | *phzD1* | *phzD1* |  |
|  |  | *phzE1* | *phzE1* | *phzE1* | *phzE1* | *phzE1* |  |
|  |  | *phzF1* | *phzF1* | *phzF1* | *phzF1* | *phzF1* |  |
|  |  | *phzG2* | *phzG2* | *phzG2* | *phzG2* | *phzG2* |  |
|  |  | *phzH* | *phzH* | *phzH* | *phzH* | *phzH* |  |
|  |  | *phzM* | *phzM* | *phzM* | *phzM* | *phzM* |  |
|  |  | *phzS* | *phzS* | *phzS* | *phzS* | *phzS* |  |
| Antiphagocytosis | Alginate biosynthesis | *alg44* | *alg44* | *alg44* | *alg44* | *alg44* |  |
|  |  | *alg8* | *alg8* | *alg8* | *alg8* | *alg8* |  |
|  |  | *algA* | *algA* | *algA* | *algA* | *algA* |  |
|  |  | *algC* | *algC* | *algC* | *algC* | *algC* |  |
|  |  | *algD* | *algD* | *algD* | *algD* | *algD* |  |
|  |  | *algE* | *algE* | *algE* | *algE* | *algE* |  |
|  |  | *algF* | *algF* | *algF* | *algF* | *algF* |  |
|  |  | *algG* | *algG* | *algG* | *algG* | *algG* |  |
|  |  | *algI* | *algI* | *algI* | *algI* | *algI* |  |
|  |  | *algJ* | *algJ* | *algJ* | *algJ* | *algJ* |  |
|  |  | *algK* | *algK* | *algK* | *algK* | *algK* |  |
|  |  | *algL* | *algL* | *algL* | *algL* | *algL* |  |
|  |  | *algX* | *algX* | *algX* | *algX* | *algX* |  |
|  | Alginate regulation | *algP* | *-* | *-* | *algP* | *algP* |  |
|  |  | *algQ* | *algQ* | *algQ* | *algQ* | *algQ* |  |
|  |  | *algR* | *algR* | *algR* | *algR* | *algR* |  |
|  |  | *algU* | *algU* | *algU* | *algU* | *algU* |  |
|  |  | *algW* | *algW* | *algW* | *algW* | *algW* |  |
|  |  | *algZ* | *algZ* | *algZ* | *algZ* | *algZ* |  |
|  |  | *mucA* | *mucA* | *mucA* | *mucA* | *mucA* |  |
|  |  | *mucB* | *mucB* | *mucB* | *mucB* | *mucB* |  |
|  |  | *mucC* | *mucC* | *mucC* | *mucC* | *mucC* |  |
|  |  | *mucD* | *mucD* | *mucD* | *mucD* | *mucD* |  |
|  |  | *mucE* | *mucE* | *mucE* | *mucE* | *mucE* |  |
|  |  | *mucP* | *mucP* | *mucP* | *mucP* | *mucP* |  |
| Biosurfactant | Rhamnolipid biosynthesis | *rhlA* | *rhlA* | *rhlA* | *rhlA* | *rhlA* |  |
|  |  | *rhlB* | *rhlB* | *rhlB* | *rhlB* | *rhlB* |  |
|  |  | *rhlC* | *rhlC* | *rhlC* | *rhlC* | *rhlC* |  |
| Enzyme | Hemolytic phospholipase C | *plcH* | *plcH* | *plcH* | *plcH* | *plcH* |  |
|  | Non-hemolytic phospholipase C | *plcN* | *plcN* | *plcN* | *plcN* | *plcN* |  |
|  | Phospholipase C | *plcB* | *plcB* | *plcB* | *plcB* | *plcB* |  |
|  | Phospholipase D | *pldA* | *pldA* | *pldA* | *-* | *pldA* |  |
|  | Pyochelin receptor | *fptA* | *fptA* | *fptA* | *fptA* | *fptA* |  |
|  | Pyochelin | *pchA* | *pchA* | *pchA* | *pchA* | *pchA* |  |
|  |  | *pchB* | *pchB* | *pchB* | *pchB* | *pchB* |  |
|  |  | *pchC* | *pchC* | *pchC* | *pchC* | *pchC* |  |
|  |  | *pchD* | *pchD* | *pchD* | *pchD* | *pchD* |  |
|  |  | *pchE* | *pchE* | *pchE* | *pchE* | *pchE* |  |
|  |  | *pchF* | *pchF* | *pchF* | *pchF* | *pchF* |  |
|  |  | *pchG* | *pchG* | *pchG* | *pchG* | *pchG* |  |
|  |  | *pchH* | *pchH* | *pchH* | *pchH* | *pchH* |  |
|  |  | *pchI* | *pchI* | *pchI* | *pchI* | *pchI* |  |
|  |  | *pchR* | *pchR* | *pchR* | *pchR* | *pchR* |  |
|  | Pyoverdine receptors | *fpvA* | *fpvA* | *fpvA* | *fpvA* | *fpvA* |  |
|  | Pyoverdine | *pvdA* | *pvdA* | *pvdA* | *pvdA* | *pvdA* |  |
|  |  | *pvdD* | *pvdD* | *pvdD* | *-* | *pvdD* |  |
|  |  | *pvdE* | *pvdE* | *pvdE* | *pvdE* | *pvdE* |  |
|  |  | *pvdF* | *pvdF* | *pvdF* | *pvdF* | *pvdF* |  |
|  |  | *pvdG* | *pvdG* | *pvdG* | *pvdG* | *pvdG* |  |
|  |  | *pvdH* | *pvdH* | *pvdH* | *pvdH* | *pvdH* |  |
|  |  | *pvdI* | *-* | *pvdI* | *pvdI* | *-* |  |
|  |  | *pvdJ* | *-* | *pvdJ* | *pvdJ* | *pvdJ* |  |
|  |  | *pvdL* | *pvdL* | *pvdL* | *pvdL* | *pvdL* |  |
|  |  | *pvdM* | *pvdM* | *pvdM* | *pvdM* | *pvdM* |  |
|  |  | *pvdN* | *pvdN* | *pvdN* | *pvdN* | *pvdN* |  |
|  |  | *pvdO* | *pvdO* | *pvdO* | *pvdO* | *pvdO* |  |
|  |  | *pvdP* | *pvdP* | *pvdP* | *pvdP* | *pvdP* |  |
|  |  | *pvdQ* | *pvdQ* | *pvdQ* | *pvdQ* | *pvdQ* |  |
|  |  | *pvdS* | *pvdS* | *pvdS* | *pvdS* | *pvdS* |  |
|  |  | *pvdY* | *pvdY* | *pvdY* | *pvdY* | *pvdY* |  |
| Protease | Alkaline protease | *aprA* | *aprA* | *aprA* | *aprA* | *aprA* |  |
|  | Elastase | *lasA* | *lasA* | *lasA* | *lasA* | *lasA* |  |
|  |  | *lasB* | *lasB* | *lasB* | *lasB* | *lasB* |  |
|  | Protease IV | *prpL* | *prpL* | *prpL* | *prpL* | *prpL* |  |
| Quorum sensing | Acylhomoserine lactone synthase | *hdtS* | *hdtS* | *hdtS* | *hdtS* | *hdtS* |  |
|  | N-(3-oxo-dodecanoyl)-L-homoserine lactone QS system | *lasI* | *lasI* | *lasI* | *lasI* | *lasI* |  |
|  |  | *lasR* | *lasR* | *lasR* | *lasR* | *lasR* |  |
|  | N-(butanoyl)-L-homoserine lactone QS system | *rhlI* | *rhlI* | *rhlI* | *rhlI* | *rhlI* |  |
|  |  | *rhlR* | *rhlR* | *rhlR* | *rhlR* | *rhlR* |  |
| Regulation | GacS/GacA two-component system | *gacA* | *gacA* | *gacA* | *gacA* | *gacA* |  |
|  |  | *gacS* | *gacS* | *gacS* | *gacS* | *gacS* |  |
| Secretion system | Hcp secretion island-1 encoded type VI secretion system (H-T6SS) | *clpV1* | *clpV1* | *clpV1* | *clpV1* | *clpV1* |  |
|  |  | *fha1* | *fha1* | *fha1* | *fha1* | *fha1* |  |
|  |  | *hcp1* | *hcp1* | *hcp1* | *hcp1* | *hcp1* |  |
|  |  | *icmF1* | *icmF1* | *icmF1* | *icmF1* | *icmF1* |  |
|  |  | *ppkA* | *ppkA* | *ppkA* | *ppkA* | *ppkA* |  |
|  |  | *pppA* | *pppA* | *pppA* | *pppA* | *pppA* |  |
|  |  | *vgrG1* | *vgrG1* | *vgrG1* | *vgrG1* | *vgrG1* |  |
|  | P. aeruginosa TTSS translocated effectors | *exoS* | *-* | *-* | *exoS* | *-* |  |
|  |  | *exoT* | *exoT* | *exoT* | *exoT* | *exoT* |  |
|  |  | *exoU* | *exoU* | *exoU* | *-* | *exoU* |  |
|  |  | *exoY* | *-* | *-* | *exoY* | *-* |  |
|  | P. aeruginosa TTSS | *exsA* | *exsA* | *exsA* | *exsA* | *exsA* |  |
|  |  | *exsB* | *exsB* | *exsB* | *exsB* | *exsB* |  |
|  |  | *exsC* | *exsC* | *exsC* | *exsC* | *exsC* |  |
|  |  | *exsD* | *exsD* | *exsD* | *exsD* | *exsD* |  |
|  |  | *exsE* | *exsE* | *exsE* | *exsE* | *exsE* |  |
|  |  | *pcr1* | *pcr1* | *pcr1* | *pcr1* | *pcr1* |  |
|  |  | *pcr2* | *pcr2* | *pcr2* | *pcr2* | *pcr2* |  |
|  |  | *pcr3* | *pcr3* | *pcr3* | *pcr3* | *pcr3* |  |
|  |  | *pcr4* | *pcr4* | *pcr4* | *pcr4* | *pcr4* |  |
|  |  | *pcrD* | *pcrD* | *pcrD* | *pcrD* | *pcrD* |  |
|  |  | *pcrG* | *pcrG* | *pcrG* | *pcrG* | *pcrG* |  |
|  |  | *pcrH* | *pcrH* | *pcrH* | *pcrH* | *pcrH* |  |
|  |  | *pcrR* | *pcrR* | *pcrR* | *pcrR* | *pcrR* |  |
|  |  | *pcrV* | *pcrV* | *pcrV* | *pcrV* | *pcrV* |  |
|  |  | *popB* | *popB* | *popB* | *popB* | *popB* |  |
|  |  | *popD* | *popD* | *popD* | *popD* | *popD* |  |
|  |  | *popN* | *popN* | *popN* | *popN* | *popN* |  |
|  |  | *pscB* | *pscB* | *pscB* | *pscB* | *pscB* |  |
|  |  | *pscC* | *pscC* | *pscC* | *pscC* | *pscC* |  |
|  |  | *pscD* | *pscD* | *pscD* | *pscD* | *pscD* |  |
|  |  | *pscF* | *pscF* | *pscF* | *pscF* | *pscF* |  |
|  |  | *pscG* | *pscG* | *pscG* | *pscG* | *pscG* |  |
|  |  | *pscH* | *pscH* | *pscH* | *pscH* | *pscH* |  |
|  |  | *pscI* | *pscI* | *pscI* | *pscI* | *pscI* |  |
|  |  | *pscJ* | *pscJ* | *pscJ* | *pscJ* | *pscJ* |  |
|  |  | *pscK* | *pscK* | *pscK* | *pscK* | *pscK* |  |
|  |  | *pscL* | *pscL* | *pscL* | *pscL* | *pscL* |  |
|  |  | *pscN* | *pscN* | *pscN* | *pscN* | *pscN* |  |
|  |  | *pscO* | *pscO* | *pscO* | *pscO* | *pscO* |  |
|  |  | *pscP* | *pscP* | *pscP* | *pscP* | *pscP* |  |
|  |  | *pscQ* | *pscQ* | *pscQ* | *pscQ* | *pscQ* |  |
|  |  | *pscR* | *pscR* | *pscR* | *pscR* | *pscR* |  |
|  |  | *pscS* | *pscS* | *pscS* | *pscS* | *pscS* |  |
|  |  | *pscT* | *pscT* | *pscT* | *pscT* | *pscT* |  |
|  |  | *pscU* | *pscU* | *pscU* | *pscU* | *pscU* |  |
|  | T6SS(Aeromonas) | *hcp1* | *-* | *-* | *hcp1* | *-* |  |
| Toxin | Exotoxin A (ETA) | *toxA* | *toxA* | *toxA* | *toxA* | *toxA* |  |
|  | Hydrogen cyanide production | *hcnA* | *hcnA* | *hcnA* | *hcnA* | *hcnA* |  |
|  |  | *hcnB* | *hcnB* | *hcnB* | *hcnB* | *hcnB* |  |
|  |  | *hcnC* | *hcnC* | *hcnC* | *hcnC* | *hcnC* |  |
